# Therapeutic potential of tumor treating fields for malignant brain tumors

**DOI:** 10.1002/cnr2.1813

**Published:** 2023-03-29

**Authors:** Youyou Zhou, Xiaoqing Xing, Jinyun Zhou, Han Jiang, Peili Cen, Chentao Jin, Yan Zhong, Rui Zhou, Jing Wang, Mei Tian, Hong Zhang

**Affiliations:** ^1^ Department of Nuclear Medicine and PET Center The Second Affiliated Hospital of Zhejiang University School of Medicine Hangzhou Zhejiang China; ^2^ Institute of Nuclear Medicine and Molecular Imaging Zhejiang University Hangzhou Zhejiang China; ^3^ Key Laboratory of Medical Molecular Imaging of Zhejiang Province Hangzhou Zhejiang China; ^4^ Faculty of Science and Technology, Department of Electrical and Computer Engineering, Biomedical Imaging Laboratory (BIG) University of Macau Taipa Macau SAR China; ^5^ Human Phenome Institute Fudan University Shanghai China; ^6^ College of Biomedical Engineering and Instrument Science Zhejiang University Hangzhou Zhejiang China; ^7^ Key Laboratory for Biomedical Engineering of Ministry of Education Zhejiang University Hangzhou Zhejiang China

**Keywords:** alternating electric field, cancer therapy, glioblastoma, malignant brain tumors, tumor treating fields

## Abstract

**Background:**

Malignant brain tumors are among the most threatening diseases of the central nervous system, and despite increasingly updated treatments, the prognosis has not been improved. Tumor treating fields (TTFields) are an emerging approach in cancer treatment using intermediate‐frequency and low‐intensity electric field and can lead to the development of novel therapeutic options.

**Recent Findings:**

A series of biological processes induced by TTFields to exert anti‐cancer effects have been identified. Recent studies have shown that TTFields can alter the bioelectrical state of macromolecules and organelles involved in cancer biology. Massive alterations in cancer cell proteomics and transcriptomics caused by TTFields were related to cell biological processes as well as multiple organelle structures and activities. This review addresses the mechanisms of TTFields and recent advances in the application of TTFields therapy in malignant brain tumors, especially in glioblastoma (GBM).

**Conclusions:**

As a novel therapeutic strategy, TTFields have shown promising results in many clinical trials, especially in GBM, and continue to evolve. A growing number of patients with malignant brain tumors are being enrolled in ongoing clinical studies demonstrating that TTFields‐based combination therapies can improve treatment outcomes.

## 1 INTRODUCTION

Malignant brain tumors are lethal diseases of the central nervous system and pose a serious threat to human health because of the specificity of tumor sites. Among them, glioblastoma multiforme (GBM) is the most common and deadliest primary neoplasm with an incidence of 5/100000 inhabitants/year and a recurrence rate approaching 100%.^1^, ^2^ The current standard of care (SOC) for GBM is multidisciplinary treatment approaches, such as the Stupp protocol, involving extensive surgery, chemotherapy and radiotherapy, the prognosis remains discouraging, showing a median survival of 15 months and a 5‐year survival rate of less than 5%.^3^ Temozolomide (TMZ) is an indispensable clinical first‐line chemotherapeutic agent for the treatment of GBM but is limited by drug resistance.^4^ The conventional approaches provide limited effectiveness for malignant brain tumors due to late diagnosis, unspecific location, and tumor heterogeneity, leading to unbearable adverse effects.^5^, ^6^ Targeted therapy and immunotherapy (e.g., gefitinib and trastuzumab) have shown modest efficacy, because of the poor permeability of the blood–brain‐barrier (BBB).^7^, ^8^, ^9^ Therefore, it's urgent to develop a more effective and safer therapy.

TTFields are a novel tumor therapy that inhibits the proliferation and growth of the tumor by delivering alternating electrical field (EF) at low intensity (1–3 V/cm) and intermediate frequency (100–300 kHz).^10^ TTFields therapy has achieved satisfactory outcomes in several clinical trials. As a physical local therapy, TTFields do not cause serious systemic side effects and are free of therapeutic resistance.^11^, ^12^ Up to now, TTFields have already been approved by the US Food and Drug Administration (FDA) for the treatment of patients with newly diagnosed glioblastoma (ndGBM), recurrent glioblastoma (rGBM), and malignant pleural mesothelioma,^11^, ^12^, ^13^ and recommended by many domestic and international expert consensus and guidelines.

Afterward, breakthroughs have been made in the in vitro or animal studies on the biological mechanisms of TTFields, and the potential of TTField‐based combination therapies continues to be explored. Clinicians have carried out some significant trials of TTFields, including combined treatments or triple treatment, making TTFields a promising candidate in combination with other therapies. In this article, we will review the anti‐tumor mechanism of TTFields and discuss the emerging framework of TTFields‐based combination therapy for malignant brain tumors.

## 2 ANTI‐TUMOR MECHANISMS OF TTFIELDS

Tissues and cells possess endogenous EF that influence biological activities and cellular events. Bioelectrical signaling regulates many essential processes to cellular homeostasis, and the biological circuitry of cancer cells is modified. Such as the processes of tumor metastasis, which can be regulated by cellular ion channels.^14^ When potassium ion channels are overexpressed in tumor cells, more negative charges will be carried inside the cells, and the imbalance of voltage will lead to an increase in tumor growth and metastasis. Manipulating the voltage characteristics of breast cancer cells can significantly reduce the number of metastatic sites in the lungs of mice by about 50%.^15^ Exogenous EF has been long exploited for interference and/or stimulation of certain natural biological processes, such as depolarization of nerves, contraction of muscles, embryonic development and heat production of tissues.^16^ Neither heat is generated nor action potentials are triggered, so the application of mid‐frequency alternating EF is neglected.^17^, ^18^ Emerging studies have reported that TTFields can alter the bioelectrical state of macromolecules and organelles involved in cancer biology, thus showcasing the therapeutic potential against tumors. Moreover, the whole proteomic and transcriptomic analyzes proved the massive alteration of differentially expressed proteins, mRNAs, miRNAs, lncRNAs, and circRNAs by TTFields in GBM cells, which are related to cell mitosis‐related events, varied cellular biological processes, and multiple organelle structures and activities.^19^

### 2.1 TTFields interfere with cancer cell mitosis

**FIGURE 1:**
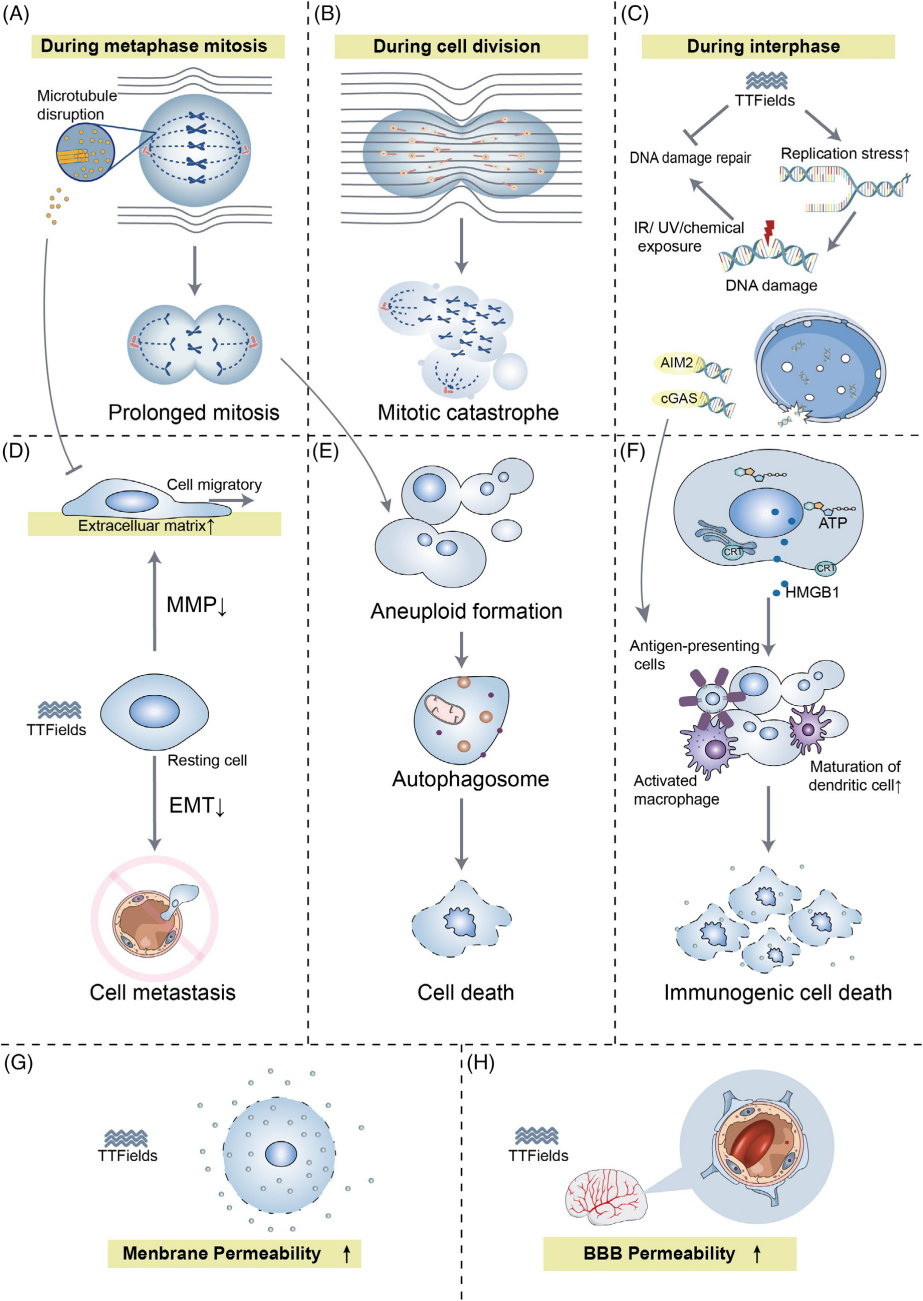
Diagram of the anti‐cancer mechanism of TTFields. (A) During metaphase mitosis, tubulin will align in the direction of the EF, resulting in interference with its polymerization and thus impaired chromosome segregation. (B) During cell division, the cell morphology under TTFields (hourglass‐like shape) produces an inhomogeneous intracellular EF, and all polar macromolecules and organelles will be pushed toward the CCF. In addition to interfering with mitosis, TTFields have multiple biological mechanisms, including (C) inhibiting DSB repair, enhancing DNA replication pressure, (D) inhibiting tumor cell migration and metastasis, (E) promoting autophagy, and (F) inflammatory responses to kill tumor cells, (G) increasing cell membrane and (H) BBB permeability to facilitate the uptake of agents.

The inhibition of cell mitosis is the most commonly reported mechanism of TTFields and TTFields target cancer cells through unusual electrical polarity and rapid proliferative properties. Considering that all charged particles and dipoles in the cell will respond to EF/currents and will oscillate as EF forces alternate in opposite directions,^20^ and dividing cells contain highly polar, spatially oriented microtubules and septins, TTFields are capable of interfering with cell mitosis and leading to the arrest of proliferation.^10^ Specifically, during normal metaphase, tubulins are precisely choreographed and arranged to form microtubule spindles that extend into the genetic material lining the center of the cell and bind to chromosomes.^21^, ^22^ When exposed to TTFields, tubulin is forced to align along the direction of EF, resulting in the interference of tubulin polymerization and obstruction of microtubule spindle formation (Figure 1A).^10^

Another important basis for the effect of TTFields in dividing cells is the directional, hourglass‐shaped cell morphology during the cytokinesis phase.^10^ At the late stage of cell mitosis, the mitotic septin complex composes of septin 2, 6, and 7, which will be repositioned in alternating EF, leading to aberrant localization of the cytokinetic cleavage furrow (CCF), which results in improper cell division.^23^ Septins cross‐link to F‐actin bundles within the submembrane actin cytoskeleton and must be sufficiently stiff to withstand the hydrostatic pressure generated within the cytoplasm by the invasion of CCF.^24^, ^25^ In cytokinesis, the applied EF intensity in the dividing cell shows an hourglass‐like non‐uniform field distribution, and all polar macromolecules and organelles will be pushed toward the CCF where the EF intensity is highest (Figure 1B).^26^, ^27^, ^28^ This so‐called “mesoelectrophoresis” prevents cells from dividing properly. Catastrophic mitotic errors that occur in late mitosis are impossibly rectified, and the cell membrane will rupture and blister.^29^ Then cancer cells will undergo cell death or apoptosis. A systematic comparison of changes in cell number and clonogenic survival of 10 solid tumor‐derived cell lines after 72 h of TTFields exposure yielded that all cell lines exhibited a decrease in cell number and clonogenic survival.^27^ However, the mechanism of apoptosis induced by TTfields has not been elucidated completely. Some studies revealed that TTFields‐induced cell death occurs in a caspase 3‐dependent manner,^30^ while some argued that TTFields‐induced apoptosis is not dependent on caspase 3.^31^

### 2.2 TTFields disrupt genomic integrity

During interphase, TTFields possess the function of disrupting the integrity of the genome, leading to efficient cytocidal actions against tumor cells. The phosphorylation level of γ‐H2AX, an indicator of DNA damage, was higher when combining TTFields with radiation treatment (RT) than RT alone.^32^ RT‐induced cytotoxicity depends on the extent of DNA double‐strand breaks (DSBs) repair, most of which are repaired within 24 h after RT. In TTFields‐treated cells after RT, more than 40% of DSB failed to be repaired, indicating the efficacy of TTFields to enhance RT‐damaged DNA may be through blocking the homologous recombination repair pathway.^33^ The expression of BRCA1 pathway genes was found significantly down‐regulated during TTFields treatment.^34^ The BRCA1 pathway not only plays a vital role in homologous recombinant DNA repair,^35^ but maintains the stability of DNA replication forks in association with the Fanconi anemia proteins and promotes alternative end‐joining DNA repair.^36^ Therefore, DNA replication stress is increased by TTFields, including the reduction of replication fork speed and the increase in R‐loop formation, resulting in the disruption of DNA integrity.^37^ In turn, TTFields not only slow DNA damage repair kinetics but also induce replication stress in cancer cells, resulting in cell death (Figure 1C).

### 2.3 TTFields inhibit cell migration and invasion

TTFields have been reported with the capacity to inhibit the metastatic spread of solid tumors.^38^ Tumor metastasis is a multi‐step process, including tumor cell invasion of basement membranes and movement to surrounding tissues, intravasation into blood vessels, and spreading to other organ sites. It's well known that microtubules in TTFields‐treated cells tend to align with the EF. Alterations of microtubules lead to the mediation of the GEF‐H1/RhoA/ROCK signaling pathway and the consequent formation of focal adhesions and induction of peripheral actin bundling, thereby hindering the motility of cancer cells (Figure 1D).^23^ TTField also exert the suppression of ciliogenesis in GBM cell lines, which is related to the development of tumor and resistance to therapy.^39^

Epithelial‐mesenchymal transition (EMT) programs promote the acquisition of aggressive properties by enhancing the motility of cancer cells, damaging the intercellular junctions, and remodeling the extracellular matrix (ECM).^40^ EMT‐related biomarkers in GBM cells were found significantly affected by TTFields involving a series of potential mechanisms. Remarkably, mesenchymal markers (e.g., vimentin, smooth muscle actin) were down‐regulated, while epithelial markers (e.g., the adherens junction protein E‐cadherin) were up‐regulated, and its loss serves as the core role in the loss of epithelial differentiation.^41^ ECM is the first tissue barrier to prevent tumor invasion peripherally, which can be degraded by matrix metalloproteinases (MMPs). TTFields can inhibit the degradation of ECM by suppressing the expression of nuclear factor kappa‐B (NF‐κB), a transcription factor that regulates the expression of MMPs.^41^, ^42^ Besides, the migration and invasion of cancers require an adequate supply of oxygen and nutrients, hence neovascularization is a decisive factor in cancer progression.^43^ The levels of hypoxia‐inducible factor 1α (HIF1α) and vascular endothelial growth factor (VEGF) were decreased in TTFields‐treated cells, leading to reduced angiogenesis.^41^ Furthermore, a significant time‐dependent inhibition in PI3K/AKT and MAPK signaling was observed in the TTFields‐treated cells, resulting in a reduction in cell migration and invasion by decreasing EMT‐ and ECM‐ related marker expression and reducing angiogenesis (Figure 1D).^41^

### 2.4 TTFields intervene autophagy process

Abnormal mitotic events can invite autophagy to occur (Figure 1E).^44^ When exposed to TTFields, the expression of autophagosome marker LC‐II/LC‐I was increased and the cells exhibited typical signs of autophagy.^31^ Akt2/mTOR/p70S6K axis (a negative regulator of autophagy) is a vital regulator of autophagy by TTFields. Moreover, many miRNAs can be induced by TTFields, especially miR‐29b which directly targets Akt2 to trigger autophagy.^45^ In most cases, autophagy can maintain the organization and stability of the centrosome to protect cells,^46^ while in the presence of the autophagy inhibitor 3‐methyladenine, the number of dead GBM cells treated with TTFields was diminished, indicating that autophagy‐mediated TTFields‐induced cell death.^31^ Such called “autophagic programmed death” may be an alternative to programmed cell death.^47^

However, inconsistent findings have been reported. Some studies proposed that autophagic flux might be interrupted by TTFields.^19^ Autophagy may be a protective mechanism of cells against TTFields in some cases. Phosphorylated AMP‐dependent kinase (AMPK) can inhibit mTORC1, thereby suppressing its ability to negatively regulate autophagy. Depletion of AMPK inhibits the up‐regulation of autophagy in response to TTFields and sensitizes GBM cells to treatment.^48^ It is unknown whether autophagy exerts a protective or killing effect on TTFields‐treated cancer cells and may be relevant to the genetic traits of cancer cells, which remains to be further investigated.

### 2.5 TTFields induce an anti‐tumor immune response

Systemic anti‐tumor immune responses can be activated in TTFields‐treated tumor‐bearing animals (Figure 1F).^38^ High doses of dexamethasone (a steroid used to relieve inflammation) could interfere with the therapeutic effect of TTFields on rGBM.^49^ In TTFields‐treated cells, damage‐related molecular patterns including high‐mobility group box 1 (HMGB1) and ATP were released, and calreticulin was exposed on the cell surface leading to increased infiltration of antigen‐presenting cells into the tumor site. Endoplasmic reticulum stress induced by TTFields may be the trigger that drives exposure of calreticulin to the cell surface.^50^ The dying cells release ATP, which serves as a “find me” signal for apoptotic cells to increase the recruitment of lymphocytes to induce immunogenic cell death.^51^ Furthermore, cell death caused by TTFields can promote the maturation and phagocytosis of dendritic cells.^50^

Emerging evidence suggested that TTFields fostered the activation of RAW 264.7 macrophages and its output of NO and ROS. When co‐cultured with 4TI cells (a breast cancer cell line) under TTFields, macrophages secreted elevated levels of pro‐inflammatory cytokines, like IL‐1β, TNF‐α, and IL‐6, and the viability of 4TI cells was diminished. Besides, the phosphorylation levels of IκB‐α, NF‐κB p65 subunit, and p38 MAPK were observed higher in TTFields‐treated RAW 264.7 cells than in control, indicating that TTFields induced the p38 MAPK/NF‐κB pathway of macrophages to exert inflammatory effects.^52^

Recently, TTFields were reported to cause local disruption of the nuclear envelope of cancer cells during interphase, resulting in the release of large cytoplasmic clusters of naked micronuclei, which recruited and activated two major DNA sensors (cGAS and AIM2). Subsequently, the activated cGAS/STING inflammasome tended to up‐regulate pro‐inflammatory cytokines, type 1 interferons (T1IFNs) and T1IFN‐responsive genes, thereby activating the peripheral immune system and creating a potential intrinsic immune platform for cancers^53^ (Figure 1C).

### 2.6 TTFields increase cell membrane permeability

When applying high‐pulsed EF to the cells, depending on the field intensity, irreversible electroporation, induction of necrotic cell death, or reversible electroporation may occur.^54^ In TTFields‐treated glioblastoma cells, the number and size of membrane pores were reversibly increased, allowing greater permeability to substances as large as 20 kDa (e.g., 5‐aminolevulinic acid) but not exceeding 50 kDa (Figure 1G).^55^ Interestingly, the effect was tumor‐specific and did not appear on normal cells. EF‐induced transient increase in plasma membrane permeability, with contributions from structural rearrangement of lipids and protein changes, causing fatigue of the membrane structure.^54^ Since the membrane composition of cancer cells is altered and more deformable than normal cells that may help in explaining the inconsistent response to alternating EF.^54^ The cell membrane permeability of tumor cells increases in response to TTFields, allowing transmembrane transport of chemical agents that explain the increased efficacy of other drugs to some extent. The cell‐membrane permeabilization also contributes to the release of innate tumor antigens and activates the immune system.^56^

Ion channels in the cell membrane can be affected by alternating EF, and the L‐type Ca^2+^ channels CACNA1C (Cav1.2) were identified as TTFields target recently,^57^ thereby influencing cell cycle progression, cell migration, and clonogenic survival of GBM cells.^58^ Cell membrane potential is a key factor controlling the switching on and off of ion channels in cell membranes, and has been identified as a target for TTFields. Employing the Schwan equation (used to calculate transmembrane potential); it was found that in tumor cells, TTFields‐induced changes in cell membrane potential can be much higher than 10% of the resting cell membrane potential, thus affecting intracellular ion homeostasis.^59^

TTFields were also reported to disrupt tight junction proteins (e.g., claudin 5 and ZO‐1) of the BBB to increase the permeability of the brain (Figure 1H).^60^ Till now, the presence of BBB has posed limitations for the treatment of brain malignancies and the cerebral application of some agents. Recently, TTFields have been proven to temporarily increase the permeability of the BBB in a human in vitro model.^61^ TTField‐induced opening of BBB is similarly reversible and expands the range of intracranial drug applications.

## 3 FACTORS AFFECTING THE EFFICACY OF TTFIELDS

The efficacy of TTFields depends on specific parameters. For different types and sites of tumors, different modalities are set to obtain optimal efficacy. The optimal intensity of EF is different for different cancer cells. Mouse melanoma cells and rat glioma cells completely stop proliferating at 1.35 and 2.25 V/cm respectively,^10^ while human non‐small cell lung cancer and breast cancer cells require higher.^26^ The frequency range for anti‐tumor effects in most cancers is 100 to 200 kHz^62^, ^63^ (e.g., 200 kHz for human GBM clinically, 120 kHz for melanoma cells^10^). In vitro studies have shown that different GBM cell lines have different optimal electrical frequencies, 200 kHz for KNS42 and GIN‐31, 400 kHz for SF18, and 100 kHz for U87.^64^, ^65^ The optimal frequency of alternating EF is inversely proportional to the size of the cancer cell.^10^ Additionally, the inhibitory effect of tumor proliferation by TTFields is time‐dependent,^27^, ^66^ and the effect of EF is strongest when the direction of EF is parallel to the axis of cell division. Given that the polarity of tumor cells during the division cycle is irregular, applying EF in different directions is an effective way to increase treatment efficacy for patients.^10^ Sequential application of multiple EF directions every 0.25 ~ 1.00 s can make the splitting axis of more cells parallel to the direction of EF.^26^

## 4 CLINICAL STUDIES OF TTFIELDS IN MALIGNANT BRAIN TUMORS

With an in‐depth understanding of the mechanism of action of TTFields, translational studies accompanying clinical trials are crucial for TTFields and are being vigorously pursued. The results of published clinical scientific research on TTFields therapy in brain malignancies have been summarized in Table 1. The ongoing clinical trials to further evaluate the safety and efficacy of TTFields‐based combination therapy have been listed in Table 2.

**TABLE 1:** The summary of finished clinical studies of TTFields in brain tumors.

Study	Phase	Cancer type	Number of patients	The device, frequency, intensity, and exposure time of TTFields	Combining therapy	Outcomes	Refs.
EF‐02	Pilot study	rGBM	1	NovoTTF‐100A device; Frequency: 100–200 kHz; Field intensity of 0.7 V/cm root mean square (RMS).	—	No response	67
EF‐07	Pilot clinical trial	rGBM	10	Not available.	—	Median time to disease progression: 26.1 weeks; Mean OS: 62.2 weeks	26
EF‐11	Randomized phase III clinical trial	rGBM	TTFields arm: 120; TMZ arm: 117	NovoTTF‐100A; Frequence: 200 kHz; Field intensity:>0.7 V/cm at the center of the brain.	—	Mean OS: 6.6 vs. 6.0 months; Mean PFS: 2.2 vs. 2.1 months.	11
—	Retrospective Study	rGBM	TMZ/BEV/IRI/TTFields arm: 18; BEV/TTFields arm: 30	NovoTTF‐100A; Exposure time: >18 h/day.	TMZ BEV, Irinotecan	Mean OS: 18.9 months; Mean PFS: 10.7 months.	68
OptimalTTF‐1	Pilot study	rGBM	15	Optune; Frequence: 200 kHz; a peak‐to‐peak current of 1.8–2 A.	Skull remodeling surgery	Mean OS: 15.5 months; Mean PFS: 4.6 months.	69
EF‐14	Randomized phase III clinical trial	ndGBM	TMZ/TTFields arm: 466; TMZ arm: 229	NovoTTF‐100A; Frequency: 200 kHz; Exposure time: ≥18 h/day.	TMZ	Mean OS: 19.6 vs. 16.6 months； Mean PFS: 7.1 vs. 4.0 months.	12
—	Clinical trial	newly diagnosed MGMT promoter methylated glioblastoma	CCNU/TMZ/TTFields≥8 weeks arm: 22 CCNU/TMZ/TTFields<8 weeks arm: 48	Not available.	CCNU, TMZ	Mean PFS: 21.5 vs. 11.2 months； Mean OS: not reached.	70
—	Retrospective Study	ndGBM	16	Optune; ≥18 h/day	TMZ, Lomustine	Mean PFS: 20 months.	71
—	Pilot study	ndGBM	30	NovoTTF‐200A; Frequency: 200 kHz; Exposure time: ≥18 h/day.	Concurrent radiotherapy, TMZ.	Mean PFS: 9.3 months.	72
—	Pilot study	ndGBM	10	NovoTTF‐200A; Frequency: 200 kHz; Exposure time: ≥18 h/day.	Radiotherapy, TMZ	Mean PFS: 8.9 months.	73

Abbreviations: BEV, Bevacizumab; GBM, glioblastoma; ndGBM, newly‐diagnosed glioblastoma; OS, overall survival; PFS, progression‐free survival; rGBM, recurrent glioblastoma; TMZ, Temozolomide.

**TABLE 2:** The summary of ongoing clinical trials of TTFields in brain tumors.

NCT number	Condition or disease	The device, frequency, intensity, and exposure time of TTFields	Combining therapy	Status
NCT02831959	Non‐small cell lung cancer brain metastasis	NovoTTF‐200A; 150 kHz; >18 h/day	Stereotactic radio surgery	Recruiting
NCT05341349	Melanoma brain metastases	NovoTTF‐100 M; ≥8 h/day	Ipilimumab, Nivolumab, Pembrolizumab, Stereotactic, Radiosurgery	Not yet recruiting
NCT04967027	Brain metastasis with drug/radiation resistant	ASCLU‐300 TTF; ≥18 h/day	—	Recruiting
NCT04221503	GBM	Optune; ≥18 h/day	Niraparib, Planned surgical resection	Recruiting
NCT04689087	rGBM	Not mentioned	Second‐line chemotherapy treatment	Recruiting
NCT04492163	rGBM	Optune; 200 kHz	—	Recruiting
NCT04223999	First recurrent GBM	≥18 h/day	Skullremodeling surgery	Recruiting
NCT04397679	ndGBM	≥18 h/day	Partial Brain radiotherapy, Temozolomide, Chloroquine	Recruiting
NCT03405792	ndGBM	Optune; 24 months	Temozolomide, Pembrolizumab	Active, not recruiting
NCT03223103	ndGBM	Optune	Poly‐ICLC, Peptides	Active, not recruiting
NCT04671459	GBM and rGBM	Optune	Stereotactic radiosurgery	Recruiting
NCT05030298	Malignant Glioma	Not available	RT, TMZ, radiosurgery	Not yet recruiting
NCT04902586	Glioma and GBM	Low‐intensity; 200 kHz; ≥18 h/day	Radiotherapy	Not yet recruiting
NCT04396860	Gliosarcoma and MGMT‐unmethylated GBM	NovoTTF‐100A	Ipilimumab, Nivolumab	Recruiting
NCT03033992	Pediatric malignant glioma ependymoma	NovoTTF‐200A; ≥18 h/day	—	Recruiting
NCT05086497	GBM	Optune array layout mapping created from conventional/advanced MR imaging sequences	—	Not yet recruiting

*Note*: All trials can be searched on the ClinicalTrials.gov beta website (https://clinicaltrials.gov/).

### 4.1 Recurrent glioblastoma

Defined as a grade IV glioma, GBM has a high recurrence rate, and there is no consensus for the optimal treatment of rGBM.^74^, ^75^ Bevacizumab (BEV), lomustine, carmustine and carmustine wafers are FDA approved for rGBM. GBM diffusely infiltrates the brain and rarely metastasizes extracranially, thus is amenable to TTFields therapy with complete coverage of the brain volume.^76^ The pilot trial of TTFields therapy was on 10 patients with rGBM, and the median time to disease progression was 26.1 weeks and the mean overall survival (OS) was 62.2 weeks, higher than the reported medians of historical control patients.^26^ In the randomized phase III clinical trial (EF‐11, NCT00379470), 237 patients with rGBM were enrolled into two arms: TTFields versus best physician's choice chemotherapy (including BEV, irinotecan, nitrosurea, carboplatin and TMZ). The results showed that OS and progression‐free survival (PFS) of patients in two arms were similar (6.6 vs. 6.0 months, 2.2 vs. 2.1 months, respectively; *p* > .05).^11^ Although no improvement was observed in the EF‐11 trial, the better quality of life (QoL) and lighter toxicity favored the TTFields when compared with chemotherapy as an option in the maintenance phase. Of note, most of the recruited patients were in the advanced stages of the disease (failure of more than 2 chemotherapy agents), and there was heterogeneity in the patient population. In the OptimalTTF‐1 trial (NCT02893137), the combination of cranial remodeling surgery with TTFields and BPC in patients with rGBM yielded an OS of 15.5 months and PFS of 4.6 months,^69^ much higher than that of TTFields arm in EF‐11, and was well tolerated by patients.^11^ The removal of a standard craniotomy bone flap increased the EF intensity at the tumor site and was more effective with multiple smaller burr holes than with a single craniectomy.^69^ Since the new multimodal approach has shown a preliminary survival benefit, a large‐scale randomized, controlled trial, phase 2 trial (OptimalTTF‐2, NCT0422399) was initiated 2 years ago to valid the novel combination therapy.^77^

### 4.2 New diagnosed glioblastoma

The SOC for ndGBM is maximal surgical resection, 6 weeks of postoperative RT and TMZ, and 6 months of TMZ maintenance therapy.^78^ In a phase III trial of ndGBM (EF‐14, NCT00916409), 695 patients after completion of chemoradiotherapy were enrolled into two arms randomly. Compared with standard TMZ maintenance therapy alone, adding TTFields to standard TMZ maintenance therapy dramatically improved OS (19.6 vs. 16.6 months, respectively; *p* < .001) and PFS (7.1 vs. 4.0 months, respectively; *p* < .001) of patients.^12^ Excitingly enough, the DNA repair protein O^6^‐methyl‐guanine DNA methyltransferase (MGMT), which has previously been reported to affect the efficacy of TMZ, did not affect TTFields.^31^ EF‐14 is the landmark trial of TTFields therapy, however, the lack of a sham device control group in this clinical trial may have led to a placebo effect. Moreover, the patient selection in EF‐14 that enrolled patients after standard chemoradiotherapy rather than at diagnosis may lead to some bias, and the timing of TTFields therapy still needs to be determined again. The NOA‐09/CeTeG trial published recently has verified the feasibility of a combination of lomustine and TMZ.^71^ Therefore, the combination of TTFields, lomustine and TMZ during the maintenance phase was tested, and the observed PFS was 20 months in 16 patients with MGMT promoter methylated ndGBM that might suggest the potential benefit of the triple maintenance therapy.^71^ A new publishment reported that the combination of TTFields and TMZ plus CCNU can provide additional survival benefits for newly diagnosed MGMT promoter methylated glioblastoma patients. Median PFS of 14.4 months and median OS of 33.8 months were observed in 70 patients.^70^

TTFields are a promising candidate radiosensitizer which induced an abnormal increase in mitotic catastrophe and DNA damage of cells.^32^ A prospective, single‐arm study that recruited 10 patients with ndGBM was conducted to assess the feasibility and safety of combined RT and TTFields therapy with maintenance TMZ and TTFields. The mean PFS of this trial was 8.9 months and limited toxicity was reported.^73^ When TTFields were given concurrently with RT which was delivered through the TTFields arrays and with maintenance TMZ, the PFS of 9.3 months was illustrated.^72^ It follows that the feasibility of TTFields both as maintenance therapy and alongside chemoradiotherapy is endorsed by prior clinical practice, and a more large‐scale clinical trial, EF‐32(NCT04471844), is underway.

### 4.3 Other gliomas

The experience with the application of TTFields to other gliomas is limited. A published clinical case for the first time reported that TTFields‐based combination therapy delayed pathological up‐gradation from anaplastic astrocytoma (a WHO grade III glioma) to GBM and prolonged PFS to nearly 10 months.^79^ This case indicated the potential benefit of TTFields‐based therapy in anaplastic astrocytoma and additional experiments of TTFields in a larger cohort of patients with anaplastic astrocytoma needed to be evaluated.

Ganglioglioma is classified as grade I, and although grows slowly, some patients will experience recurrence or malignant progression. Transformation of ganglioglioma to high‐grade glioma is rare but usually with a poor prognosis.^80^ A patient with a BRAF V600E mutation in a high‐grade glioma originating from ganglioneuroma was treated with dabrafenib (a selective inhibitor of BRAF V600E) and TTFields after the failure of TMZ‐based therapy. More than 2 years of follow‐up have shown a complete response to the combination therapy.^81^ The superiority of gene‐targeted treatment in combination with TTFields therapy should be supported by the outcomes of large‐scale, multiply cohort clinical trials in the future.

### 4.4 Metastatic tumors

The majority of brain metastases result from lung cancer, while in women the most common primary histology is breast cancer.^82^ The development of brain metastases complicates many solid tumors and is attributed to the death of patients with advanced cancer.^83^ A METIS trial enrolling non‐small cells lung cancer (NSCLC) patients with 1–10 brain metastases (NCT02831959) who receive stereotactic radiosurgery (SRS) followed by TTFields (150 kHz, ≥18 h/day) or supportive care within 7 days of SRS reported no safety issues in 2018 and is ongoing currently.^84^

## 5 PRECLINICAL STUDIES OF TTFIELDS IN MALIGNANT BRAIN TUMORS

With an optimal output pattern, TTFields could significantly inhibit the viability, proliferation, and invasiveness of different cell lines, irrespective of their genetic traits.^85^ Several studies have shown synergistic effects of TTFields and targeted agents, of which BEV is widely applied.^68^, ^86^ Sorafenib is a multi‐kinase inhibitor and a first‐line agent for the treatment of high‐grade gliomas as well, but it has failed to improve the outcomes of patients when combined with TMZ.^87^ While combined with TTFields, sorafenib significantly inhibited motility, invasiveness, and angiogenesis of GBM cells.^88^ Evolutionary conserved protein kinase monopolar spindle 1 (MPS1) inhibition also has an impact on the mitotic process, resulting in more than an additive effect when combined with TTFields to GBM cells, and the anti‐proliferative benefit of the combination therapy begins to work earlier than mono‐therapy.^89^ Some inhibitors of MPS1 have been developed and can potentially serve as a bridge for TTFields therapy interruption. Likewise, hyperthermia has been reported to increase the efficacy of other approaches against cancers. Combining heat therapy with TTFields has been reported to enhance each other's therapeutic effects and inhibit the metastasis of GBM cells.^90^ The combination of two physical therapy may be easily tolerated by patients. Since Ca^2+^ channels contribute to cellular stress response to TTFields, combining TTFields with Ca^2+^ antagonists (e.g., benidipine) may augment the efficacy and outcome of TTFields.^57^ It provides the possibility of combining TTFields with Ca^2+^ antagonists, which are already applied in clinical. In brief, TTFields hold great promise to address the challenge across the spectrum of the management of patients with high‐grade gliomas by optimizing other treatment strategies.

Given the special immune environment of intracranial tumors, brain malignancies suppress immune cell activity and anti‐cancer function. Revitalizing the central immune system has become the emerging for malignant brain tumors and experienced tremendous growth.^5^ Considering the transformation of tumors under TTFields exposure to a state more favorable for an anti‐tumor immune response,^53^ and its role of switching on the BBB, TTFields may potentially enhance the central anti‐tumor immune response.^60^, ^91^ At present, immunotherapeutic approaches for GBM contain vaccines, checkpoint inhibitors, CAR‐T cells, and oncolytic viruses.^5^ Combining TTFields and anti‐PD‐1 (programmed cell death protein 1) therapy has already been proven more valid in the resistance to extra‐cranial tumors than single therapy by triggering immune response.^50^ Several clinical trials (e.g., NCT03223103, NCT03405792) to test the immunogenicity and safety of immunotherapeutic approaches in combination with TTFields in patients with malignant brain tumors are conducted currently, which will be valuable to establish a combination of therapeutic strategies and to elucidate the mechanism of TTFields on the immune micro‐environment of malignant brain tumors.

## 6 THERAPEUTIC EVALUATION OF TTFIELDS THERAPY

Magnetic resonance imaging (MRI) is the common method for diagnostics and follow‐up of malignant brain diseases, especially the change of MRI contrast enhancement, which is often considered an indicator of treatment response or tumor progression.^92^ But conventional MRI cannot assess treatment response reliably due to a lack of specificity,^93^ and the enhancement can be caused by other non‐tumor‐associated processes.^94^ Physiological imaging techniques, including diffusion tensor imaging (DTI), dynamic susceptibility contrast‐perfusion weighted imaging (DSC‐PWI) and proton MR spectroscopy, have great potential in assessing treatment response to different therapies in patients with GBM. A case reported the experience in evaluating treatment response to TTFields in combination with TMZ in a female ndGBM patient using DTI, DSC‐PWI and 3D‐echo‐planar spectroscopic imaging. Compared with baseline, increased mean diffusivity and decreased fractional anisotropy, maximum relative cerebral blood volume, and reduced choline/creatine were noted at 2 months follow‐up periods.^95^ The MRI results verified the synergistic effect of TTFields and TMZ chemotherapy can inhibit the growth of tumors.

**FIGURE 2:**
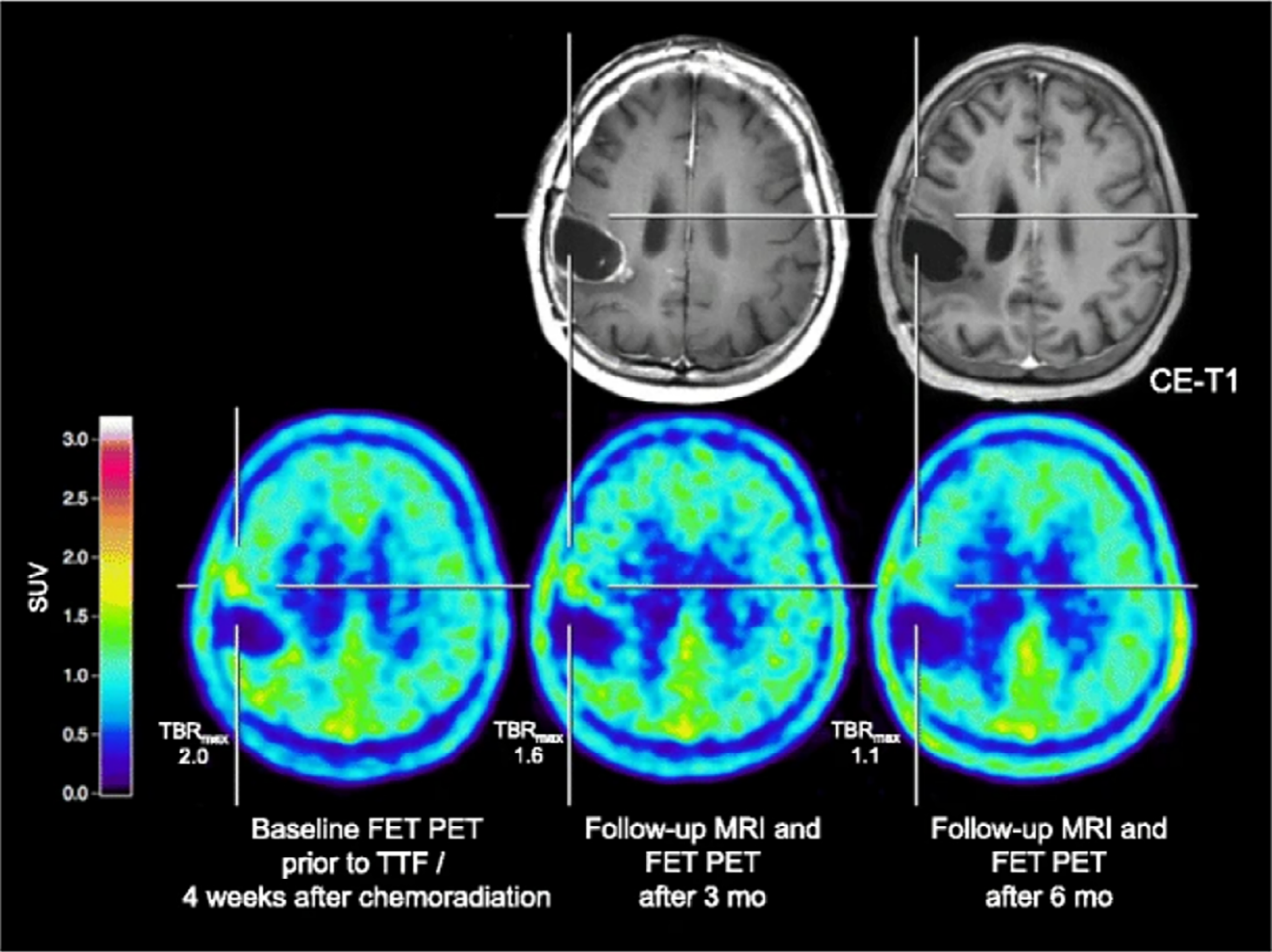
Images before TTFields and during follow‐up of a 73‐year‐old patient with GBM. Due to immune thrombocytopenia, only TTFields were applied 4 weeks after completion of radiotherapy with concomitant TMZ. Before TTFields, baseline FET PET images showed a slight increase in metabolic activity (TBRmax 2.0, TBRmean 1.6) without spatial counterpart contrast enhancement. Sequential FET PET images at 3 and 6 months follow‐up showed a decrease in metabolic activity, as evidenced by a decreased tumor‐to‐brain ratio. Compared to MRI T1 images, FET PET images showed clearer lesions. Adapted permission from (Ref. 100).

Positron emission tomography (PET) scanning provides more biological information than just anatomical information in a non‐invasive way.^96^ As the most widely clinically applied tracer, ^18^F‐FDG(^18^F‐2‐fluoro‐2‐deoxy‐D‐glucose) can be highly taken up by normal brain tissue, thus limiting the application in patients with malignant brain tumors.^97^ The Response Assessment in Neuro‐Oncology group has recommended more wide‐scale diagnostic access to amino acids‐based PET for the management of patients with malignant brain tumors,^98^ due to the low uptake by normal brain tissue.^99^ When FET (O‐(2‐^18^F‐fluoroethyl)‐L‐tyrosine) PET scanning was applied in patients with high‐grade glioma whose treatment included TTFields, the data showed increased tumor volume with increased uptake or metabolic activity and demonstrated with histologically or clinically follow‐up confirmed disease progression. Compared to MRI images, FET PET images present clearer lesion boundaries (Figure 2).^100^ AMT (alpha[C‐11]‐methyl‐L‐tryptophan) PET scanning has been proven to detect an early metabolic response in rGBM patients before and after TTFields therapy. The images indicated that AMT PET can detect the metabolic alterations of amino acids in GBM cells induced by TTFields earlier than MRI, therefore helping in clinical decision‐making, especially in cases where MRI images are inconclusive.^101^ ^18^F‐DASA‐23 (1‐((2‐fluoro‐6‐^18^F‐fluorophenyl)sulfonyl)‐4‐((4‐methoxyphenyl)sulfonyl)piperazine) was introduced to provide the information on the level of pyruvate kinase M2 (PKM2),^102^ which is preferentially expressed in cancer cells and contributes to anabolic glucose metabolism.^103^ A research project on manipulating ^18^F‐DASA‐23 radiotracer to detect the impairment of GBM glycolytic metabolism through down‐regulation of the expression of PKM2 by TTFields was published.^104^ This research proved that TTFields can cause a shift in GBM metabolism from glycolysis to oxidative phosphorylation for the first time. Even in the presence of sufficient oxygen, cancer cells prefer to use the inefficient process of glycolysis for energy production,^105^ the so‐called Warburg effect, which produces lactic acid that is beneficial for tumor growth and metastasis.^106^ Warburg effect attributes to the tumor progression and provides a suitable and appropriate atmosphere for tumor to metastasize. Therefore, the metabolic reprogramming of tumor cells by TTFields may also be one of its significant anti‐cancer mechanisms.

## 7 TTFIELDS IMPROVE THE QUALITY OF PATIENT'S LIFE

Till now, accumulating clinical experience has confirmed the safety of TTFields therapy, and no significantly life‐threatening TTFields‐related event has been reported.^11^, ^12^ The most frequent TTFields‐related side effect is skin reaction,^107^ while other therapies often cause serious systematic side effects. The array‐associated skin toxicity including rash, irritation, erythema, pruritus, contact dermatitis, ulceration, infection and skin breakdown, could be cured with topical corticosteroid creams, topical and oral antibiotics, and isolation of the affected skin surface from adhesives or pressure.^108^ In brief, TTFields are linked to prolonged survival outcomes, minimal adverse effects and are easily manageable.

According to the patient registration dataset, the clinical efficacy of TTFields on patients was significantly improved with ≥75% daily adherence (≥18 h/day) compared with <75%.^109^ In other words, the efficacy of TTFields on patients with brain malignancies critically depends on their high compliance rate. Patients must abide by a long daily regimen of continuous TTFields for at least 4 weeks, which might be inconvenient for patients, allowing patients to receive treatment by simply wearing the devices on their heads and not interfering with their daily activities. The most commonly employed TTFields delivery system consists of four transducer arrays, a field generator, and a power resource. The distribution of the arrays can be determined by the NovoTAL software to ensure that the best field strength is obtained at the tumor site.^110^ EF is delivered through the transducer arrays attached to the skin, and patients with lesions in the brain need to make an adequate commitment to daily skin care, regular hair shaving, and carrying sufficient power sources during TTFields therapy. Patients are more likely to experience the psychological burden of changes in appearance, and the inconvenience of wearing the arrays and carrying the battery packs for several hours a day. Fortunately, according to the analysis of the EF‐14 trial, adding TTFields to cancer treatment does not worsen patient function and well‐being production, such as role, social, and physical functioning.^107^ Patients can enjoy symptomatically stability during TTFields therapy, meanwhile, their quality of life can be maintained.

Since late 2019, the COVID‐19 epidemic has forced a change in the treatment paradigm brought about by quarantine limitations, which might become a barrier to the routine treatment of patients with cancer. TTFields can be administered via telemedicine and confer a competitive advantage to be conveniently employed for the management of GBM patients during the COVID‐19 pandemic.^111^ Furthermore, reducing patient visits to healthcare facilities and cutting down on potential exposure to the virus will ensure the safety of ongoing treatment.

## 8 CONCLUSION

As a noninvasive physical therapy, TTFields have exhibited unique advantages in the treatment of patients with malignant brain tumors and are well suited for clinical trials exploring combined approaches to the management of patients due to the minor adverse effects and absence of toxicity. Nevertheless, the application of TTFields in pediatric high‐grade glioma is relatively rare, with few case reports regarding safety analysis, and needs to be corroborated in a larger patient cohort.^112^ TTFields do not have a half‐life and enable sustainable treatment,^113^ which can be applied between chemoradiotherapy or concurrently with RT.^114^ Furthermore, TTFields are inherently non‐specific to tumor types, may cause therapeutic effects for a wide range of solid tumors, and are being investigated for application to extracranial tumors.^50^, ^63^, ^115^ Unfortunately, the mechanism of TTFields is far less clear and suffers from skepticism than other therapies.^116^ Pharmacological profiling may help reveal the synergistic effects of TTFields in combination with other therapeutic agents. Continued enhancement of research and understanding of the molecular mechanisms will facilitate the adoption of this novel therapy for integration into existing or new treatment strategies.

Although already become an established treatment for ndGBM or rGBM in adults and has been proven effective in combination with SOC of GBM, TTFields were not recommended to be added to SOC of GBM by the leading experts.^114^ High cost is the biggest obstacle to broadening the application of TTFields, which needs to be addressed immediately, and additional cost‐effectiveness studies are recommended.^117^, ^118^ Therefore, improving the efficiency of TTFields will greatly benefit cancer treatment and reduce global medical costs. The phase II clinical trial EF‐33 (NCT04492163) for rGBM is being carried out, which applies higher field strength to the tumor by increasing the number of electric field arrays under the condition of safety assurance to achieve a better therapeutic effect. To improve the convenience of use, further refinement of the TTFields system is required to improve the ease of use and device performance through physical and other means (e.g., changing the size and weight of the instrument, implanting electrodes around the tumor, and increasing battery capacity). Conducting an outpatient clinic for TTFields therapy consultation is suggested, which may lead to a great promotion of motivation and compliance rate, and ensures the clinical efficacy of TTFields.^119^ Besides, it's still a mission for researchers to figure out the biomarkers to select the suitable patients who might be responsive to TTFields, and the markers or technique predicting optimal frequency or response in different cancer types or individuals. The best combination formula based on TTFields for special populations should also be defined. The framework of TTFields therapy, from patient selection and treatment to the follow‐up efficacy assessment system, needs to be refined in detail. As an innovative treatment with great potential for tumor therapy, we firmly believe that the TTFields treatment system will be a boon to patients with cancer.

## AUTHOR CONTRIBUTIONS

**Xiaoqing Xing:** Writing – original draft (supporting). **Jinyun Zhou:** Writing – review and editing (lead). **Han Jiang:** Writing – review and editing (supporting). **Peili Cen:** Writing – review and editing (supporting). **Chentao Jin:** Writing – review and editing (supporting). **Yan Zhong:** Writing – review and editing (supporting). **Rui Zhou:** Writing – review and editing (equal). **Hong Zhang:** Conceptualization (equal); funding acquisition (equal); supervision (equal); validation (equal).

## FUNDING INFORMATION

This work was supported by the National Natural Science Foundation of China (81725009, 21788102 and 82030049), and the National Key R&D Program of China (2021YFE0108300).

## CONFLICT OF INTEREST STATEMENT

The authors have stated explicitly that there are no conflicts of interest in connection with this article

## ETHICS STATEMENT

Not applicable.

## Data Availability

Not applicable.
